# SARS-CoV-2 Myocarditis Due to Severe Obesity

**DOI:** 10.7759/cureus.15074

**Published:** 2021-05-17

**Authors:** Aneeba Farooqi, Nayha Tahir, Om Parkash, Grace W Ying, Farah Zahra

**Affiliations:** 1 Internal Medicine, Chicago Medical School Internal Medicine Residency Program at Northwestern Medicine McHenry Hospital, McHenry, USA

**Keywords:** obesity, obesity-related illnesses, covid, myocarditis, covid-induced myocarditis

## Abstract

Myocarditis is defined as a myocardial injury concomitant with myocardial dysfunction. Several causes are associated with it, including infectious versus inflammatory and inherited cardiomyopathies. It can be acute, subacute, or chronic, and it can present as focal versus diffuse myocardial dysfunction. Viruses diseases, including the Coxsackie B3 virus, have been a well-established cause of viral myocarditis. It is a significant cause of mortality typically among young individuals due to lymphocytic or granulomatous inflammation of the myocardium. At present, severe acute respiratory syndrome coronavirus 2 (SARS-CoV-2) has been a detrimental cause of myocarditis with significant mortality and morbidity. Literature has revealed that most of the individuals affected by SARS-CoV-2 have significant other comorbidities, including cardiovascular, renal, or endocrine system-related comorbidities. It is noticed worldwide that patients with hypertension, diabetes mellitus, chronic obstructive pulmonary disease, and obesity are at a higher risk of developing severe infection. Obesity itself is related to chronic low-grade inflammation, and SARS-CoV-2 infection creates an environment of an inflammatory storm by excessive activation of cytokines, thus creating a vicious cycle of injury and organ damage. We present the case of a 33-year-old Hispanic morbidly obese male without other comorbidities diagnosed with SARS-CoV-2 pneumonia, complicated by severe systolic heart failure due to SARS-CoV-2 myocarditis.

## Introduction

The ongoing pandemic caused by severe acute respiratory syndrome coronavirus 2 (SARS-CoV-2) has affected more than seven million people worldwide, with initial cases reported from Wuhan in China and spreading exponentially throughout the world. Several comorbid conditions have shown worsening outcomes, including higher length of stay in an intensive care unit (ICU) and increased morbidity and mortality. Cardiovascular diseases, including coronary artery disease (CAD), hypertension (HTN), and diabetes mellitus (DM), respiratory diseases, including pulmonary fibrosis and chronic obstructive pulmonary disease (COPD), metabolic derangements, including obesity and adrenal insufficiency (AI), are among the common comorbidities. Obesity has been reported as an independent predictor of worse outcomes in previous pandemics caused by Asian and Hong Kong hemagglutinin type 1 and neuraminidase type 1 (H1N1) influenzas in 1960 and 1968. Currently, SARS-CoV-2 disease has shown worse clinical outcomes in obese individuals with body mass index (BMI) greater than 30 kg/m^2^ or severe obesity with BMI greater than 35 kg/m^2^ even in the absence of other comorbidities due to persistent low-grade inflammation and abnormal secretion of cytokines from excessive adipose tissue.

## Case presentation

**Figure 1:**
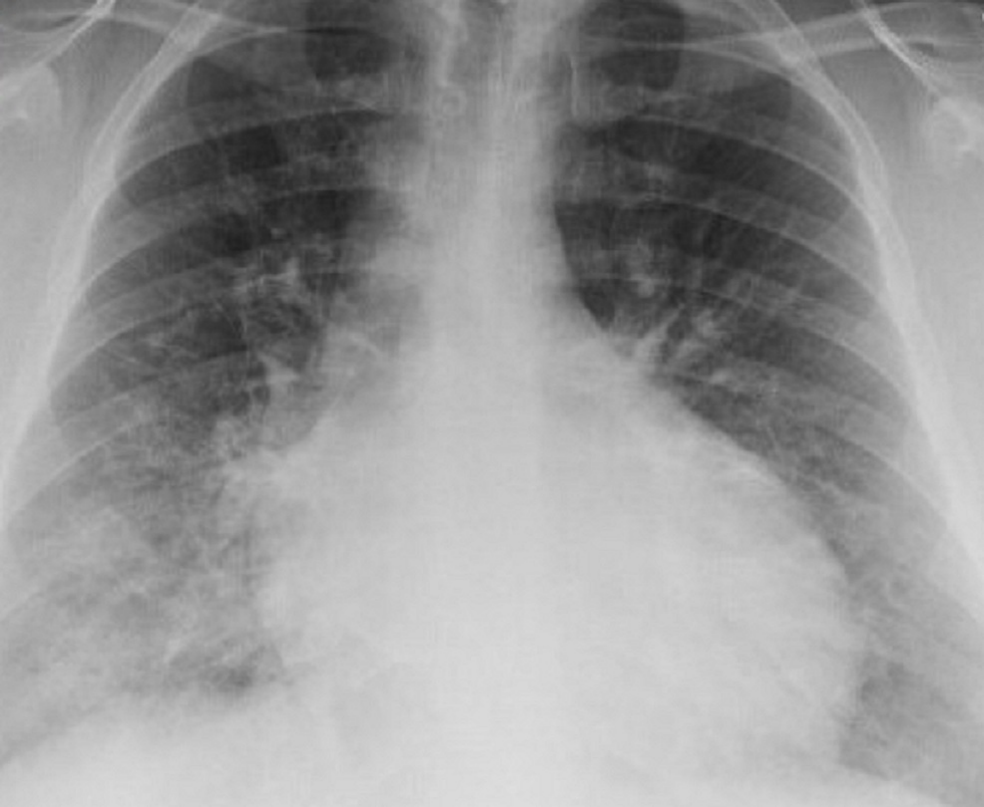
Chest X-ray showing bibasilar airspace opacities and cardiomegaly.

We present the case of a 33-year-old morbidly obese Hispanic male who presented to urgent care (UC) due to persistent shortness of breath and cough for over three weeks. At UC, his physical examination was significant for tachycardia. The patient tested positive for SARS-CoV-2 on a nasal swab. Chest X-ray showed bibasilar airspace opacities concerning multifocal pneumonia (Figure 1), and the patient was sent to the emergency department (ED) for further evaluation.

**Figure 2:**
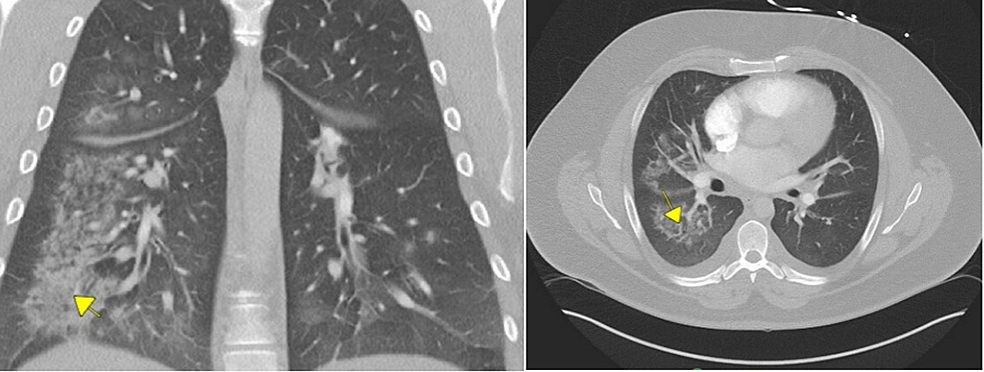
CT chest showing bibasilar airspace opacities, predominantly right-sided and prominent left ventricle enlargement (sagittal, coronal section). CT: computed tomography

Due to persistent tachycardia and dyspnea, computed tomography (CT) of the chest was done in the ED. It showed multifocal opacities with a ground-glass pattern, prominent interlobular septal lines, and pleural effusions concerning for congestive heart failure and reflux of contrast into hepatic veins and inferior vena cava with concerns of right heart dysfunction (Figure 2).

Laboratory data were remarkable for lymphocyte count of 15% (normal: 25-40%), B-type natriuretic peptide of 793 pg/mL (normal: <101 pg/mL), C-reactive protein of 1.5 mg/L (normal: <0.3 mg/L), with normal D-dimer and ferritin levels.

**Figure 3:**
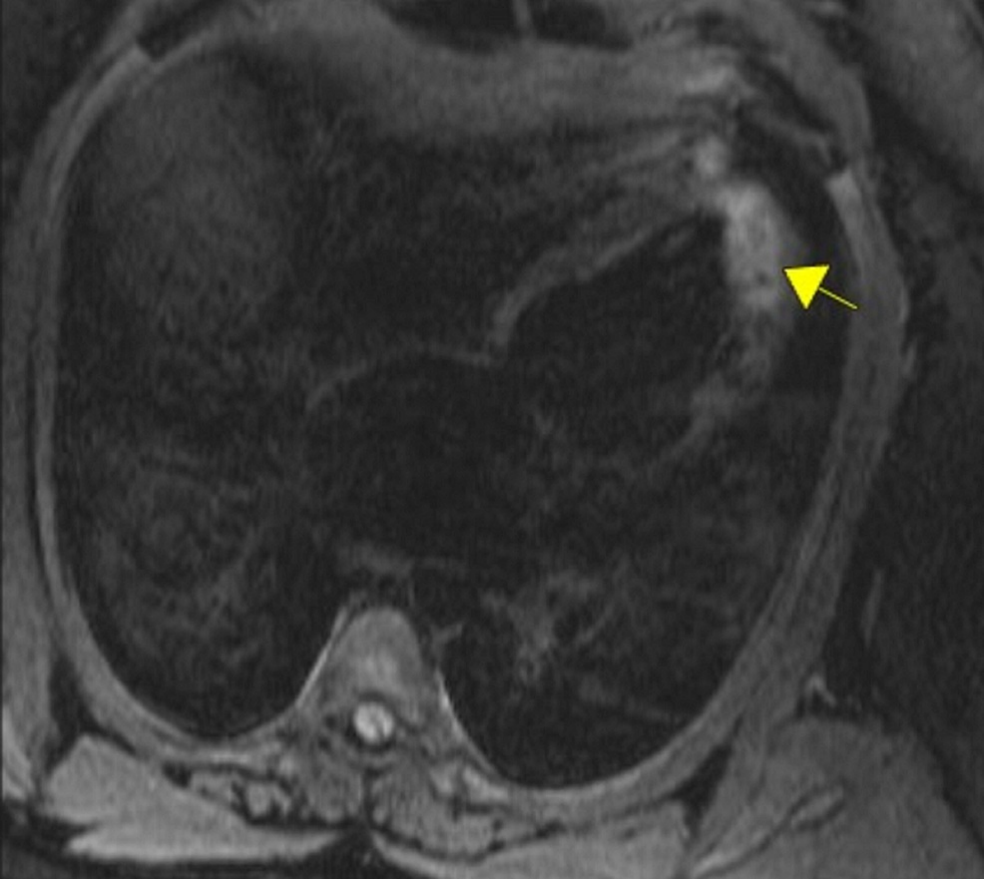
Cardiac MRI showing myocardial enhancement of left ventricle on T2 images concerning for myocardial inflammation. MRI: magnetic resonance imaging

The patient was started on remdesivir for SARS-CoV-2 and doxycycline and ceftriaxone to cover possible superimposed bacterial pneumonia. Due to concerns for right heart failure and underlying undiagnosed obstructive sleep apnea secondary morbid obesity, a transthoracic echocardiogram (TTE) was done. The results revealed some unexpected information with left ventricular ejection fraction (LVEF) of 25% without any diastolic dysfunction or valvular abnormalities. The cardiology service was consulted, and the patient was started on an optimal regimen for new-onset systolic heart failure with reduced ejection fraction. Other than morbid obesity patient did not have any other associated risk factors, including an unremarkable history of coronary artery disease in the family, thus the new-onset heart failure was presumed to be in the setting of SARS-CoV-2 infection. The patient underwent cardiac magnetic resonance imaging that showed biventricular dilation without hypertrophy and global hypokinesis, as well as left ventricular wall apical and anterolateral edema highly suggestive of myocarditis (Figure 3).

During hospitalization, the patient remained well saturated on room air. He completed a five-day course of remdesivir, and antibiotics were discontinued on day three. The patient was prescribed Coreg, Lasix, and potassium chloride and was discharged in a stable condition. He was followed up in the outpatient heart failure clinic two and four weeks post-discharge. Despite improving clinical picture, almost six weeks later, repeat TTE showed worsening LVEF of 21%. Patient diuretics were adjusted as per his clinical condition. Two weeks later the patient started to have worsening shortness of breath and lower extremity edema. He presented again to UC, was directed to ED, and was readmitted to the hospital. This time chest X-ray and CT chest showed multifocal, bilateral ground-glass opacities, and enlarged cardiac silhouette, though unchanged from the previous admission. The patient was readmitted and started on intravenous diuretics and angiotensin-converting enzyme (ACE) inhibitor. He responded appropriately initially but later got hypotension, and for a short period, diuretics and ACE inhibitor were put on hold, and the patient was started on Dobutamine infusion for more inotropic support. Overall, the patient clinically deteriorated in the next two to three days, requiring 5-6 L of oxygen from nasal cannula to heated high-flow to bi-level positive airway pressure. Around the ninth day of the second admission, the patient went into respiratory failure due to flash pulmonary edema followed by cardiorespiratory arrest and needed intubation and mechanical ventilation. The patient did achieve return of spontaneous circulation twice during the cardiopulmonary resuscitation; however, despite all heroic measures, he went into asystole. The family decided to withdraw the care and the patient passed away a couple of minutes later.

## Discussion

Like previously well-known causes of viral myocarditis affecting the myocardium, including coxsackievirus B3 [1], SARS-CoV-2 infection also affects the cardiac myocytes in various ways. The SARS-CoV-2 pandemic has impacted the entire world over the last year, starting initially in December 2019 and spreading enormously through the world. The highest hit regions, including United States, China, and Italy, have reported more than 400,000 deaths [2]. The virus has presented in a versatile manner ranging from cough, dyspnea, and fatigue to acute respiratory distress syndrome. Several studies have revealed that the most common comorbidities associated with worse clinical outcomes are HTN, DM, and obesity. Such patients require a higher level of care, including more frequent use of antibiotics, steroids, and the need for intubation and mechanical ventilation, in view of significantly elevated mortality rate in obese patients compared to other comorbidities [2,3]. Because SARS CoV-2 penetrates the human cells via binding with ACE receptors, with excessive adipose tissue, a higher number of receptors are present that potentiate the binding of the virus. The number of ACE receptors is typically higher in adipose tissues than lungs, reflecting that adipose tissues are more vulnerable to viruses than lungs. Obese individuals have higher levels of insulin resistance, more chances to get DM, and excessive activation of the renin-angiotensin-aldosterone system, potentially leading to increased vasoconstriction in the pulmonary vasculature and an overt risk of pulmonary ventilation due to perfusion mismatch. A growing body of evidence has shown immune system activity attenuation and excessive inflammation-causing endothelial dysfunction, thus promoting a proatherogenic state in obese patients that link to severe SARS-CoV-2 infection [4,5]. Data from the United States, United Kingdom, and Mexico revealed elevated mortality rates among obese SARS-CoV-2 patients, and obesity has been the second most substantial independent risk factor after age for hospitalization [6]. Comparing China, Italy, and United States, the mortality is significantly high at 65% due to 55% obese population in the United States compared to 40% in China and 20% in Italy [7]. Because abdominal obesity leads to impaired ventilation at the bases of lungs causing decreasing oxygen saturation and elevated circulating cytokines, such as interleukins, leptin, interleukin 6, and tumor necrosis factor-alpha, there is a more robust inflammatory storm, suppressed immune response, and more detrimental effects on lung parenchyma and bronchioles [8]. The prevalence of severe SARS-CoV-2 infection has been more noticeable in obese male individuals, specifically with class II obesity with a BMI greater than 35 kg/m^2^ [8]. The previous pandemic with H1N1 influenza showed increased mortality with obesity-related effects among affected individuals [9]. Finally, such individuals potentially have fewer effects of vaccination due to decreased innate and adaptive immune response in the setting of severe obesity [10]. Our patient, who was morbidly obese, presented with less severe respiratory symptoms though he had significant imaging findings of pulmonary parenchyma involvement. Due to myocarditis caused by the inflammatory process and significant lung involvement, he could not fight against SARS-CoV-2 infection.

## Conclusions

Literature has shown that previously during the H1N1 influenza pandemic of 2009-2010 and now during the SARS-CoV-2 pandemic, obesity and severe obesity are linked to the disproportionate impact on clinical outcomes. It affects pulmonary function by decreasing the expiratory reserve volume, functional capacity, and respiratory system compliance. It also causes decreased diaphragmatic movements in the supine position. Furthermore, excessive adipose tissue leads to disruption of insulin and leptin signaling, resulting in impaired viral clearance and disproportionate hyperinflammatory response. Furthermore, low intake of essential nutrients compromise organ systems in obese individuals, thus possibly contributing to increased morbidity and mortality in obese SARS-CoV-2-infected patients. Obese patients tend to have more extended ICU stays and are more challenging to manage for healthcare workers. The imaging techniques are less sensitive in such patients due to higher fat content. Also, they are difficult to intubate and more cumbersome to be kept in the prone position. Clinicians should be mindful when treating adolescent and young individuals with SARS-CoV-2 and consider setting up weight loss goals if possible. Nonetheless, social isolation is further putting a heavy toll on rising obesity incidence. Our goal through this case is to bring to attention that diagnosing excessive weight, measuring anthropometric parameters, routine checking of immune markers, and early referral to centers that are qualified to give appropriate care to obese adolescents when necessary is crucial in the treatment of SARS-CoV-2-infected obese patients. Lastly, such individuals will potentially have fewer effects of vaccination due to decreased innate and adaptive immune response in the setting of severe obesity.

## References

[1] Rose NR (2016). Viral myocarditis. Curr Opin Rheumatol.

[2] Ejaz H, Alsrhani A, Zafar A (2020). COVID-19 and comorbidities: deleterious impact on infected patients. J Infect Public Health.

[3] Korakas E, Ikonomidis I, Kousathana F (2020). Obesity and COVID-19: immune and metabolic derangement as a possible link to adverse clinical outcomes. Am J Physiol Endocrinol Metab.

[4] Petrakis D, Margină D, Tsarouhas K (2020). Obesity ‑ a risk factor for increased COVID‑19 prevalence, severity and lethality (Review). Mol Med Rep.

[5] Sanchis-Gomar F, Lavie CJ, Mehra MR, Henry BM, Lippi G (2020). Obesity and outcomes in COVID-19: when an epidemic and pandemic collide. Mayo Clin Proc.

[6] Simonnet A, Chetboun M, Poissy J (2020). High prevalence of obesity in severe acute respiratory syndrome coronavirus-2 (SARS-CoV-2) requiring invasive mechanical ventilation. Obesity (Silver Spring).

[7] (2020). WHO. Global Health Obsevatory (GHO) Data: overweight and obesity. https://www.who.int/gho/ncd/risk_factors/overweight_obesity/obesity_adults/en.

[8] Dietz W, Santos-Burgoa C (2020). Obesity and its implications for COVID-19 mortality. Obesity (Silver Spring).

[9] Nogueira-de-Almeida CA, Del Ciampo LA, Ferraz IS, Del Ciampo IRL, Contini AA, Ued FDV (2020). COVID-19 and obesity in childhood and adolescence: a clinical review. J Pediatr (Rio J).

[10] Albashir AAD (2020). The potential impacts of obesity on COVID-19. Clin Med (Lond).

